# In Utero Fetal Weight in Pigs Is Regulated by microRNAs and Their Target Genes

**DOI:** 10.3390/genes12081264

**Published:** 2021-08-19

**Authors:** Asghar Ali, Eduard Murani, Frieder Hadlich, Xuan Liu, Klaus Wimmers, Siriluck Ponsuksili

**Affiliations:** 1 Leibniz Institute for Farm Animal Biology, Institute for Genome Biology, Wilhelm-Stahl-Allee 2, 18196 Dummerstorf, Germany; ali@fbn-dummerstorf.de (A.A.); murani@fbn-dummerstorf.de (E.M.); hadlich@fbn-dummerstorf.de (F.H.); liu.xuan@fbn-dummerstorf.de (X.L.); wimmers@fbn-dummerstorf.de (K.W.); 2 Faculty of Agricultural and Environmental Sciences, University Rostock, 18059 Rostock, Germany

**Keywords:** fetal growth, longissimus dorsi muscle, prenatal development, skeletal muscle, intrauterine growth

## Abstract

Impaired skeletal muscle growth in utero can result in reduced birth weight and poor carcass quality in pigs. Recently, we showed the role of microRNAs (miRNAs) and their target genes in prenatal skeletal muscle development and pathogenesis of intrauterine growth restriction (IUGR). In this study, we performed an integrative miRNA-mRNA transcriptomic analysis in longissimus dorsi muscle (LDM) of pig fetuses at 63 days post conception (dpc) to identify miRNAs and genes correlated to fetal weight. We found 13 miRNAs in LDM significantly correlated to fetal weight, including miR-140, miR-186, miR-101, miR-15, miR-24, miR-29, miR-449, miR-27, miR-142, miR-99, miR-181, miR-199, and miR-210. The expression of these miRNAs decreased with an increase in fetal weight. We also identified 1315 genes significantly correlated to fetal weight at 63 dpc, of which 135 genes were negatively correlated as well as identified as potential targets of the above-listed 13 miRNAs. These miRNAs and their target genes enriched pathways and biological processes important for fetal growth, development, and metabolism. These results indicate that the transcriptomic profile of skeletal muscle can be used to predict fetal weight, and miRNAs correlated to fetal weight can serve as potential biomarkers of prenatal fetal health and growth.

## 1. Introduction

Prenatal growth of skeletal muscles directly impacts the weight of farm animals and meat quality [1]. Birth weight is an indicator of prenatal embryonic and fetal development and is an important trait closely associated with piglet survival, growth, and carcass quality [1]. Birth weight also reflects the in utero nutrients’ availability to the growing fetus and its ability to utilize these nutrients towards growth and development [2]. Skeletal muscle is the most abundant tissue in the body, and account for nearly 45% of total body proteins in adult humans [3]. The number of skeletal muscle fibers and muscle mass is determined during the prenatal period, which further increases the importance of proper prenatal muscle development [4,5,6]. In pigs, muscle fiber formation during the prenatal period takes place in major waves. The first wave starts at around 35 days post conception (dpc) and ends by around 60 dpc, during which myoblasts start differentiating into primary myofibers [6,7]. The second wave consists of the formation of secondary myofibers using primary myofibers as a template [7]. Secondary myofibers start appearing at 55 dpc [7]; however, the exact timing of myofiber development varies with species. The third wave is postnatal, which starts at around 60 days after birth. During the third waves, no new muscle fibers are generated, but there is a transition between slow-oxidative and fast-glycolytic fibers [7].

Prenatal muscle development is regulated by molecular pathways that are not well understood. In recent years, microRNAs (miRNAs) have been the center of attention due to their regulatory roles in diverse biological processes through post-transcriptional repression and epigenetic regulation of their target genes [8,9]. Multiple genes are targeted by a single miRNA and multiple miRNAs can target a single gene, which makes miRNAs a major player in gene regulation and important biological processes, including fetal and placental development [9,10,11]. Several studies have showed the role of miRNAs and their target genes in skeletal muscle development at different prenatal and postnatal stages across different breeds of pigs [7,12,13,14,15,16,17].

Myogenesis intensity varies with the size of the fetus, and small-sized or growth-restricted fetuses have less muscle mass compared to normal fetuses, suggesting a role of impaired skeletal muscle development in the pathogenesis of intrauterine growth restriction (IUGR) [18,19]. The number and density of myoblasts and myogenesis intensity also varies with the animal species and breed. For instance, prenatal myogenesis intensity and the number of myoblasts are higher in fat pig breeds compared to lean pig breeds [7,14,20]. In addition to variation in myogenesis intensity, the expression of miRNAs and genes regulating muscle development also differs across different pig breeds. Zhao et al. performed transcriptomic analysis of skeletal muscle in two different pig breeds and showed the involvement of different sets of genes in muscle development [7]. In Tongcheng pigs, C-X-C motif chemokine ligand 10 (*CXCL10*)*,* eukaryotic translation initiation factor 2B subunit 5 (*EIF2B5*)*,* proteasome 20S subunit α 6 (*PSMA6*), and F-box protein 32 (*FBXO32*), whereas in Yorkshire pigs, sarcoglycan delta (*SGCD*), endoglin (*ENG*), thrombomodulin (*THBD*), aquaporin 4 (*AQP4*), and BTG anti-proliferation factor 2 (*BTG2*) were dominantly involved in muscle development [7]. Zhang et al. reported that miRNAs and their target gene clusters correlated to myogenesis, and myosin component and chromatin modification were differentially expressed in Landrace and Wuzhishan pig breeds [14]. In a previous study, we compared the miRNA profile of longissimus dorsi muscle (LDM) at different prenatal and postnatal stages in German Landrace (DL) and Pietrain (Pi) pig breeds, which differ in muscularity and fat content [21]. We reported that the miR-17 family and miR-17-92 cluster have higher expression in Pi pigs compared to DL pigs [21]. In a recent study, we established an F2 population by crossbreeding Pi and DL breeds and performed a transcriptomic analysis in LDM of fetuses at 63 dpc and identified novel miRNA-mRNA networks involved in pathogenesis of IUGR. We reported that 47 upregulated, 6 downregulated miRNAs, and their differentially expressed target genes in IUGR fetuses enriched several pathways and biological processes important for fetal growth and development [12]. In the current study, we used transcriptomic data from 118 F2 fetuses generated by crossbreeding of Pi and DL pigs to identify miRNAs and genes correlated with fetal weight at 63 dpc. We hypothesized that in utero fetal growth in pigs is regulated by miRNAs and their target genes. The aim of this study was to identify the miRNAs that can be used as potential biomarkers to predict fetal health and growth in pigs.

## 2. Materials and Methods

### 2.1. Animals and Sample Collection

The breeding plan of animals and study design has been previously described [12]. Briefly, an F1 population was generated by crossbreeding DL and Pi breeds. One F1 father was mated with 11 F1 dams to produce an F2 population, which contained 118 fetuses. At 63 dpc, the sows were slaughtered, fetuses were collected and weighed, and longissimus dorsi muscle (LDM) was isolated. These animals were not subjected to any experimental treatment before slaughtering. LDM tissue was snap frozen in liquid nitrogen immediately after collection, and then transferred to −80 °C. Data from 12 F2 fetuses categorized as intrauterine growth restricted (IUGR) and 12 F2 fetuses categorized as appropriate for gestational age (AGA) have been recently published as a comparative study [12]. In the current study, the transcriptomic data from LDM of all 118 F2 fetuses were analyzed to identify miRNAs and genes correlated to fetal weight at 63 dpc. Animal care and tissue collection procedures were approved by the Animal Care Committee of the State Mecklenburg-Western Pomerania, Germany (State Office Agriculture, Food Safety and Fishery; LALLF M-V/TSD/7221.3-2.1-010/03), and husbandry and slaughter conformed to the German Law of Animal Protection. The experimental protocol was approved by the Animal Care Committee of the Leibniz Institute for Farm Animal Biology, Dummerstorf, Germany.

### 2.2. RNA Isolation

Total RNA was isolated from LDM tissue using the Tri-Reagent and RNeasy Mini kit (Qiagen) with an on-column DNase treatment according to the manufacturer’s protocol. The RNA integrity was assessed on a 1% agarose gel by electrophoresis. The RNA concentration was measured by a Nano Drop ND-1000 Spectrophotometer (PEQLAB). The quality and quantity of small RNA were assessed with an Agilent 2100 Bioanalyzer (Agilent) using an Agilent small RNA kit.

### 2.3. Gene Expression Profiling

Porcine Snowball Microarray (Affymetrix) containing 47,880 probe sets was used to determine the expression profile of the LDM from 118 F2 fetuses at 63 dpc. Using the Affymetrix WT plus Expression kit and Genechip WT terminal labeling and hybridization kit according to the manufacturer’s instructions, 500 ng of total RNA isolated from each tissue sample were used for cDNA synthesis and subsequent biotin labeling. Each of the labeled cRNA samples were hybridized on the array. The hybridization, washing, and scanning of the arrays were performed in accordance with the manufacturer’s recommendations. Affymetrix GCOC1.1.1 software was used for quality control. Expression Console software was used for robust multichip average (RMA) normalization and the detection of present genes by applying the detection above background (DABG) algorithm. Further filtering was done by excluding transcripts with low signals and probe sets that were present in less than 80% of the samples. For further analyses, 11,288 probe sets passed the quality filtering and were used. The expression data are available in the Gene Expression Omnibus public repository with the GEO accession number GSE162754.

### 2.4. MicroRNA Microarray Analysis

Affymetrix customized microarrays (GEO: GPL14969) were used [22]. Targets for hybridization were prepared from miRNA with the FlashTag™ Biotin RNA Labeling Kit for Affymetrix GeneChip miRNA arrays (Genisphere, Hatfield, PA, USA) according to the manufacturer’s recommendations. Briefly, 250 ng of miRNA of each individual were poly(A)-tailed using ATP–poly-A-Polymerase, then FlashTag Biotin end-labelled. After the hybridization of biotin-labelled complementary RNA, chips were washed and processed to detect biotin-containing transcripts by Streptavidin-PE (Phycoerythrin) conjugate, then were scanned on a GeneChip scanner 3000 7G (Affymetrix, Santa Clara, US). Data were extracted from the images, and spots were quantified and processed by quality filtering. Expression Console software was used for robust multichip average (RMA) normalization and the detection of present miRNAs by applying the DABG (detection above background) algorithm. Further filtering was done by excluding probe sets that were present in less than 80% of the samples and annotated miRNAs that had a sequence greater than or equal to 30 nucleotides in length. For further analysis, 675 probe sets passed the quality filtering and were used. The expression data are available in the Gene Expression Omnibus public repository with the GEO accession number GSE162755.

### 2.5. Transcriptomes Correlated to Fetal Weight

To determine the transcripts correlated to fetal weight at 63 dpc, the normalized expression data were used. Pearson pairwise correlation between gene expression levels and fetal weight was calculated. For both miRNA and mRNA data, *p* values were adjusted according to the Benjamini–Hochberg method to control for FDR [23]. Transcripts with FDR-adjusted *p* values < 0.05 were considered as significantly correlated to fetal weight. To confirm that the correlation results were not biased by sex, we calculated the sex-influenced transcripts using a linear model (GLM procedure, SAS 9.4 software). No miRNA influenced by sex reached the significance threshold (FDR < 5%). We found no overlap of transcripts influenced by sex with the list of transcripts correlated with fetal weight (FDR < 5%).

### 2.6. Prediction of Downstream Targets of miRNAs

To determine the downstream target genes of miRNAs significantly correlated to fetal weight, 17,065 3′-UTR sequences, 16,857 5′-UTR sequences, and 20,310 coding sequences were extracted from the *Sus scrofa* (SS) genome (Sscrofa11.1) based on Ensembl annotation version 102. These sequences were fragmented into 2000 base pair fragments with a 50-base pair overlap. Using the whole mature miRNA sequence, RNAhybrid version 2.1.2 was used to predict the target genes of 13 miRNAs correlated to fetal weight by setting the parameter as for a single hit per target, human-based assumed *p*-value distribution, minimum free energy (MFE) threshold of < −25 kcal/mole, and helix constraint from base 2 to 7 [24,25]. The Pearson correlation between miRNAs and mRNAs was calculated. Only negatively correlating miRNA–mRNA pairs were used for further analyses.

### 2.7. Enrichment Analysis

In the first step, 1383 genes correlated to fetal weight were subjected to downstream gene ontology enrichment analysis for biological processes and KEGG pathway enrichment analysis using ClueGO (version 2.5.1) and Cluepedia (version 1.5.7) plugin in the Cytoscape (version.3.8.2) environment [26,27,28]. In the second step, 13 miRNAs significantly correlated to fetal weight and their negatively correlated 135 target genes were used for gene ontology enrichment analysis with a focus on biological processes and KEGG pathway enrichment analysis. The parameters used for ClueGO analysis were a hypergeometric test that was used for enrichment analysis and Benjamini–Hochberg correction was used for multiple testing correction and the SS genome assembly as a reference. ClueGO generates functionally annotated KEGG pathways and gene ontologies of a given list of genes. KEGG pathways and gene ontologies that passed *p* ≤ 0.05 were considered significantly enriched.

## 3. Results

### 3.1. Correlation between miRNA and mRNA Expression and Fetal Weight

**Figure 1:**
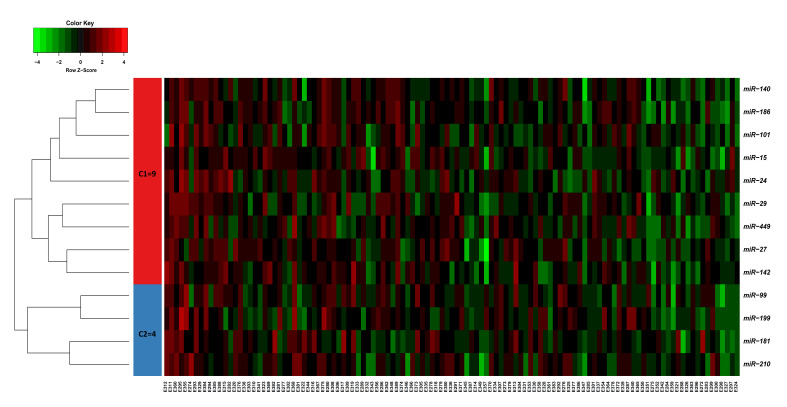
Heatmap of miRNAs significantly correlated to fetal weight (FDR < 0.05) in LDM of 118 pig fetuses at 63 dpc. Heatmap of the miRNAs expression profiles was generated using the hierarchical clustering method of heatmap.2 function of gPlots package (version 3.0.1) in the R programing environment (version 4.0.3) [29]. A total of 13 miRNAs correlated to fetal weight were distributed in two clusters based on their co-expression with cut-off criteria of variance-stabilizing transformations ≥ 2, |logFC ≥ 1|. In the color key, the red color represents high expression, the green color represents low expression, and the black color represents no change in expression.

A total of 11 F1 dams were mated with one F1 sire, all belonging to the F1 generation developed by crossbreeding DL and Pi pigs. This resulted in an F2 generation that included a total of 118 fetuses (58 males and 60 females), which were collected at 63 dpc. The weight of fetuses at 63 dpc ranged from 78.4 to 196.6 g. Litter size ranges from 5 to 15 fetuses/sow and average weight of fetuses per sow ranges from 136.8 to 169.5 g. The expression of 675 miRNA probes was calculated for the correlation with the trait of fetal weight at 63 dpc. In total, 33 miRNA probes belonging to 13 miRNA families were identified as significantly correlated to fetal weight at FDR < 0.05, including miR-140, miR-186, miR-101, miR-15, miR-24, miR-29, miR-449, miR-27, miR-142, miR-99, miR-199, miR-181, and miR-210 (Figure 1; Table S1). The heatmap for miRNAs was generated after arranging the data according to increasing fetal weight, and the 13 miRNAs significantly correlated to fetal weight were grouped in two clusters, including 9 miRNAs in cluster 1 and 4 miRNAs in cluster 2 (Figure 1). Moreover, the heatmap showed a trend of a decrease in the expression of these miRNAs with an increase in the fetal weight (Figure 1). Similarly, out of 47,880 probe-sets on the snowball microarray, 11,288 quality-filtered probes were further analyzed. The correlation between mRNA expression and fetal weight was calculated, and 1959 probes corresponding to 1315 annotate transcripts were significantly correlated to fetal weight (FDR < 0.05) at 63 dpc. All mRNA probes are shown in Table S2.

### 3.2. MicroRNA-mRNA Networks Correlated to Fetal Weight

**Figure 2:**
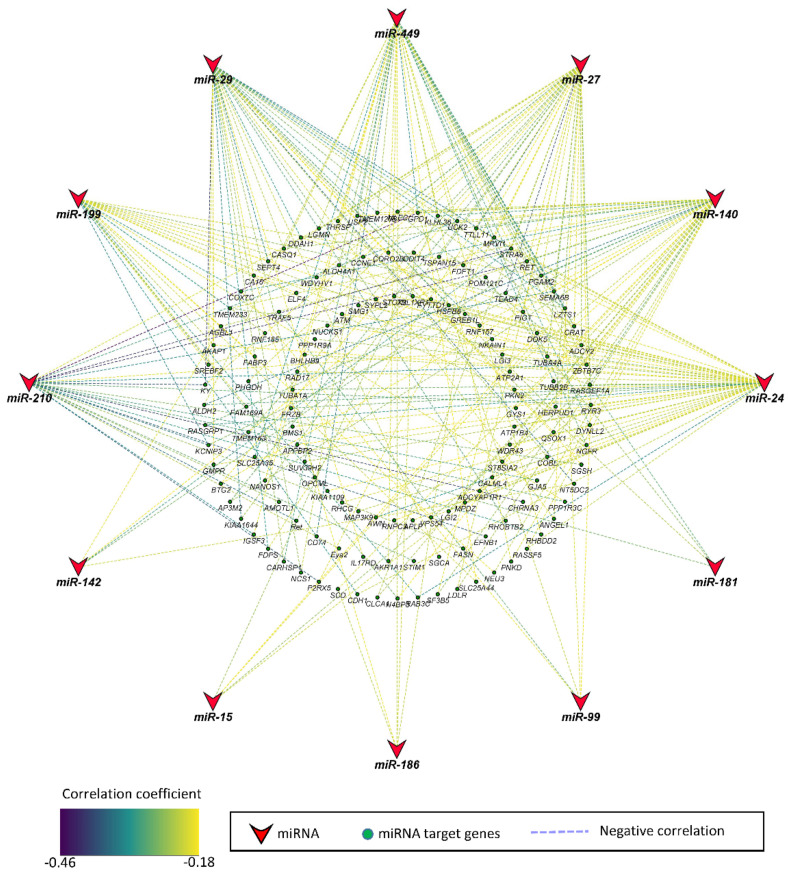
Correlation network of miRNAs and their target genes correlated to fetal weight at 63 dpc. The stroke color indicates the value of correlation coefficient according to the color key. The correlation coefficient ranged from −0.18 to −0.45.

Pearson correlation between miRNAs and their target genes significantly correlated to fetal weight was determined. Genes that were negatively correlated to miRNAs were considered significant at a threshold of 5% FDR. Out of 13 miRNAs correlated to fetal weight, 12 miRNAs were negatively correlated to 135 potential target genes, whereas no genes met the criteria of being negatively correlated and a potential target of miR-101 (Figure 2, Table S3). The correlation coefficient between miRNAs and mRNAs ranged from −0.18 to −0.45. The 12 miRNAs included miR-24, miR-140, miR-27, miR-449, miR-29, miR-199, miR-210, miR-99, miR-186, miR-15, miR-142, and miR-181, which were negatively correlated to 45, 40, 36, 35, 33, 25, 23, 5, 5, 5, 5, and 4 potential target genes, respectively (Table S3).

### 3.3. KEGG Pathway and Gene Ontology Enrichment Analysis of Genes Correlated to Fetal Weight

**Figure 3:**
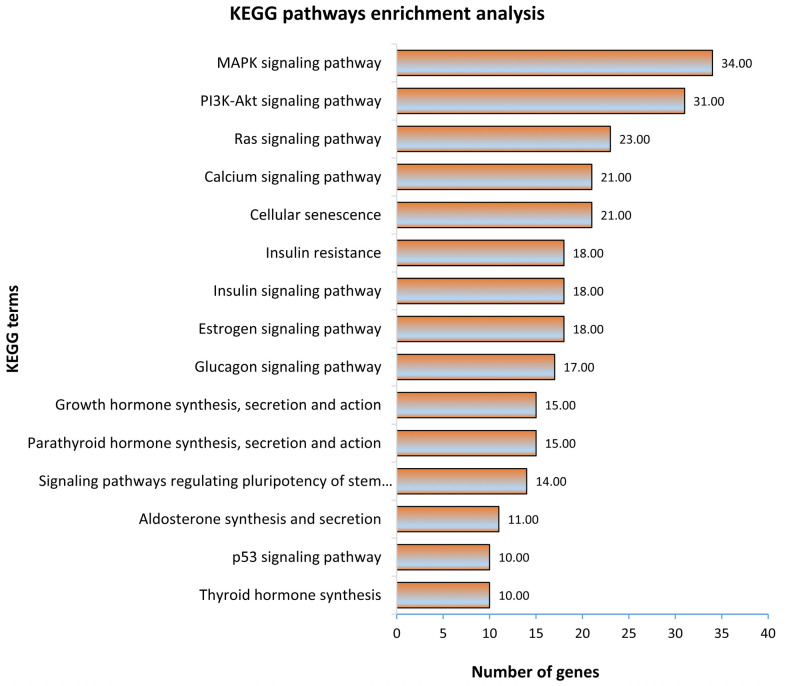
KEGG pathway enrichment analysis of genes significantly correlated to fetal weight at 63 dpc. KEGG pathways with a *p* ≤ 0.05 were considered significantly enriched.

**Figure 4:**
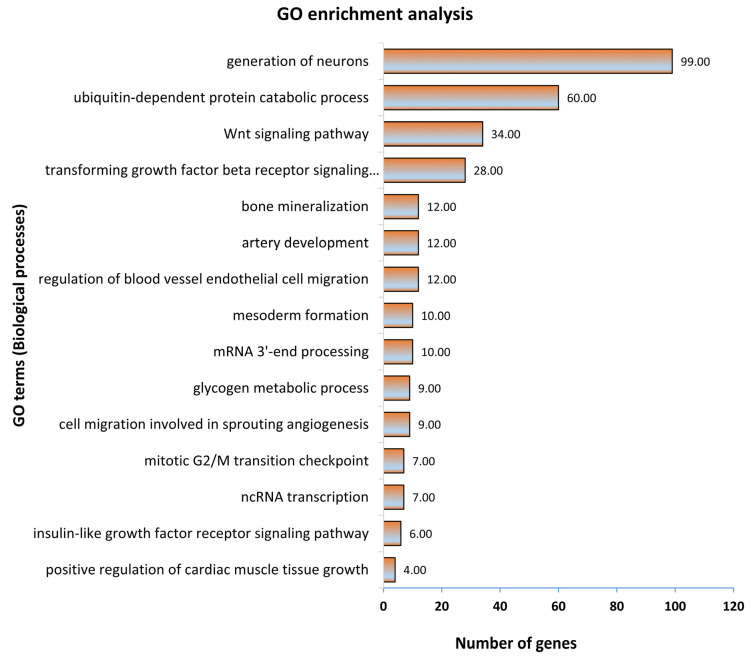
Gene ontology enrichment analysis, with a focus on biological processes, of genes significantly correlated to fetal weight at 63 dpc. Biological processes with a *p* ≤ 0.05 were considered significantly enriched.

In the first step, all 1315 genes significantly correlated to fetal weight were subjected to gene ontology and KEGG pathway enrichment analysis. The functionally annotated KEGG pathway network generated by ClueGO showed that genes significantly correlated to fetal weight enriched 110 KEGG pathways (Figure 3; Table S4a). Some significantly enriched KEGG pathways important for fetal weight and metabolism included growth hormone synthesis, secretion and action, parathyroid hormone synthesis, secretion and action, glucagon signaling pathway, thyroid hormone synthesis, estrogen signaling pathway, glucagon signaling pathway, and signaling pathways regulating pluripotency of stem cells (Figure 3). A complete list of all enriched KEGG pathways and associated genes in provided in Table S4a. Similarly, the gene ontology enrichment analysis showed that fetal weight-correlated genes significantly enriched 393 biological processes (Figure 4; Table S4b). Some of the significantly enriched biological processes important for fetal growth and development included generation of neurons, Wnt signaling pathway, bone mineralization, artery development, transforming growth factor β signaling pathway, insulin-like growth factor receptor signaling pathway, and mesoderm formation (Figure 4). A complete list of all enriched biological processes and associated genes is provided in Table S4b.

### 3.4. KEGG Pathway and Gene Ontology Enrichment Analysis of Genes Targeted by Fetal Weight-Correlated miRNAs

**Figure 5:**
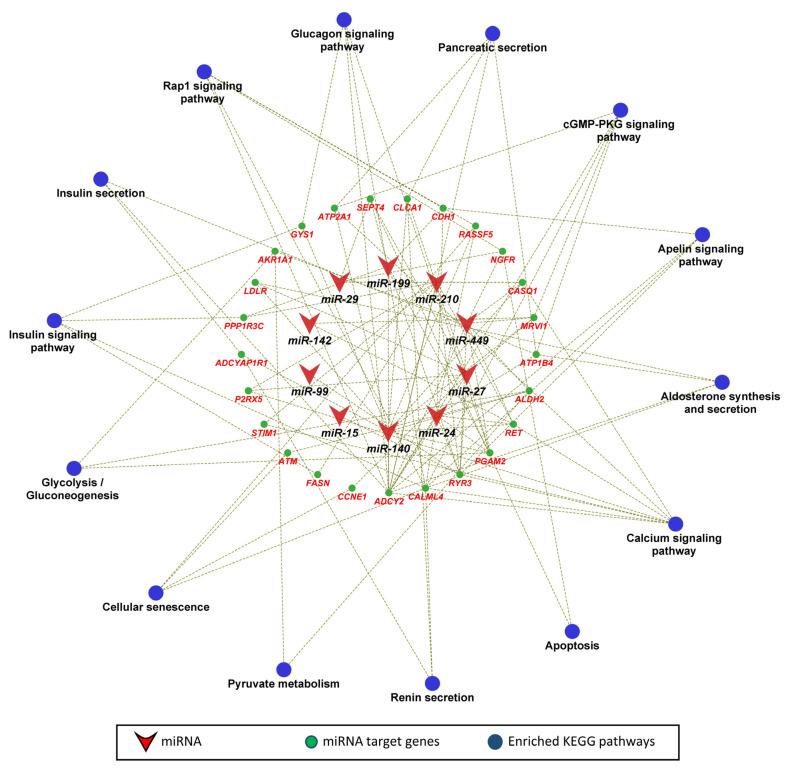
KEGG pathway enrichment analysis of fetal weight-correlated target genes of fetal weight-correlated miRNAs. KEGG pathways with a *p* ≤ 0.05 were considered significantly enriched.

**Figure 6:**
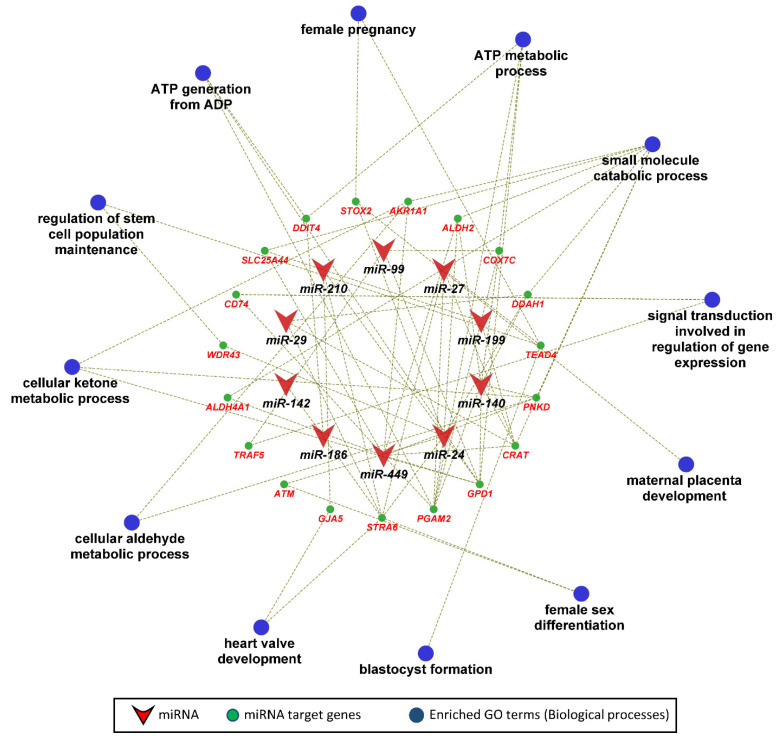
Gene ontology enrichment analysis, with a focus on biological processes, of fetal weight-correlated target genes of fetal weight-correlated miRNAs. Biological processes with a *p* ≤ 0.05 were considered significantly enriched.

In the next step, we performed KEGG pathway and gene ontology enrichment analysis for 135 genes that were negatively correlated as well as potential targets of 12 miRNAs significantly correlated to fetal weight at 63 dpc. The functionally annotated KEGG pathway network generated by ClueGO showed that miRNA target genes significantly enriched 28 KEGG pathways, including the glucagon signaling pathway, insulin secretion, insulin signaling pathway, pancreatic secretion, glycolysis/gluconeogenesis, calcium signaling, aldosterone synthesis and secretion, and pyruvate metabolism (Figure 5). Similarly, the gene ontology enrichment analysis showed that miRNA target genes significantly enriched 47 biological processes, including ATP generation from ADP, ATP metabolic process, regulation of stem cell population maintenance, female pregnancy, blastocyst formation, female sex differentiation, maternal placenta development, and heart valve development (Figure 6).

### 3.5. Fetal Weight-Correlated miRNAs and Their Potential Involvement in IUGR

**Figure 7:**
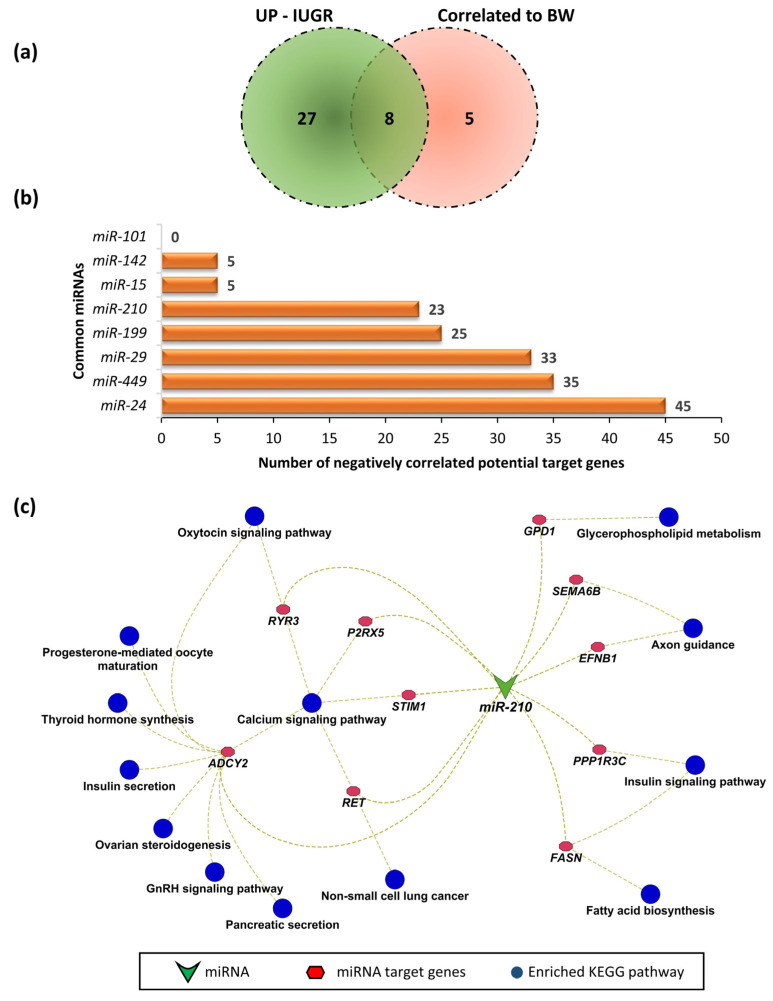
(**a**) Comparison of miRNAs correlated to fetal weight and miRNAs upregulated during IUGR. (**b**) Common miRNAs and their negatively correlated target genes. (**c**) KEGG pathway enrichment analysis of target genes of miR-210.

We previously selected 12 fetuses representing an extreme for intrauterine growth restricted (IUGR) and 12 as appropriate for gestational age (AGA) [12]. We reported that miRNAs from 33 families were upregulated in LDM from IUGR fetuses, and were potentially involved in the pathogenesis of IUGR. We further validated our findings in a larger group of samples by using 118 F2 fetuses in our recent study. We compared 13 miRNAs significantly correlated to fetal weight with the 33 miRNAs upregulated during IUGR in LDM of fetuses at 63 dpc, and found 8 common miRNAs (Figure 7a), including miR-101, miR-142, miR- 15, miR-210, miR-199, miR-29, miR-449, and miR-24, which can target 0, 5, 5, 23, 25, 33, 35, and 45 negatively correlated genes, respectively (Figure 7b). Due to evidence of involvement of miR-210 in fetal growth and development, we further investigated the KEGG pathways enriched by miR-210 target genes identified as significantly correlated to fetal weight in the current study. We found that the target genes of miR-210 enriched important KEGG pathways, including the oxytocin signaling pathway, progesterone-mediated oocyte maturation, thyroid hormone synthesis, insulin secretion, ovarian steroidogenesis, gonadotropin releasing hormone (GnRH) signaling pathway, and pancreatic secretion (Figure 7c).

## 4. Discussion

Meat is an important source of dietary proteins for human consumption and pigs are primary contributors in the meat industry. Postnatal growth and survival of piglets depends on prenatal skeletal muscle development and fetal growth, which can also influence their carcass quality [1]. A better understanding of molecular biology of prenatal skeletal muscle development can help to improve animal health as well as meat production. Different pig breeds are commercially used for meat production that vary in feed efficiency, growth rate, fat content and distribution, as well as the transcriptomic profile [30,31]. Genes are master regulators of almost all biological processes and pathways [32], hence analysis of the transcriptomic profile can be useful to better understand the growth and performance of a particular animal species or breed. In this study, we analyzed the LDM transcriptomic data of an F2 population of a pig crossbreed developed by crossbreeding of Pi and DL pigs. We found 1315 genes and 13 miRNAs in LDM that were significantly correlated to fetal weight at 63 dpc. Moreover, the expression of 13 miRNAs significantly correlated to fetal weight (miR-140, miR-186, miR-101, miR-15, miR-24, miR-29, miR-449, miR-27, miR-142, miR-99, miR-199, miR-181, and miR-210) decreased with an increase in fetal weight, indicating their role in fetal growth and skeletal muscle development.

Qin et al. performed a microRNAome analysis of pig skeletal muscle at five prenatal and five postnatal stages and identified 28 myogenic miRNAs differentially expressed across different developmental stages [17], including nine myogenic miRNAs (miR-140, miR-101, miR-24, miR-29, miR-27, miR-181, miR-186, miR-15, and miR-199) reported in the current study. In a recent study, we compared the transcriptomic profile of LDM from intrauterine growth-restricted (IUGR) fetuses and appropriate for gestational age (AGA) fetuses [12]. Eight miRNAs upregulated in LDM from IUGR fetuses (miR-101, miR-142, miR-15, miR-210, miR-199, miR-29, miR-449, and miR-24) were found to be significantly correlated to fetal weight in this study, reinforcing the importance of these miRNAs in prenatal fetal growth and skeletal muscle development. These miRNAs have a profound role in muscle growth development. miR-101 promotes satellite cell differentiation in goat skeletal muscles by targeting enhancer of zester homolog 2 (*EZH2*) [33,34], and its expression gradually increases during C2C12 myoblast differentiation [35]. miR-27 and miR-142 are major regulators of lipid utilization by skeletal muscle cells [36]. miR-27 facilitates lipid utilization and reduces glycolysis, whereas miR-142 inhibits lipid utilization by muscle fibers [36]. Upregulated miR-142 in denervated skeletal muscles targets mitofusin-1 (*MFN1*) and causes apoptosis and mitophagy, leading to denervation-induced muscle atrophy [37]. miR-15 represses the expression of SET domain containing 3 (*SETD3*) and results in repression of skeletal muscle cells [38]. miR-210 is one of the most studied hypoxia-induced miRNAs, and has a well-described role in fetal development and pregnancy complications like preeclampsia and IUGR [39]. In C2C12 myoblast cells, miR-210 expression increases upon myogenic differentiation and its activation in differentiated cells is dependent on hypoxia-inducible factor 1-α (*Hif1a*) [40]. Our results showed that miR-210 is negatively correlated and can potentially target 23 genes significantly correlated to fetal weight at 63 dpc. The miR-210 target genes enriched important pathways like fatty acid biosynthesis, thyroid hormone signaling, insulin signaling, glycerophospholipid metabolism, and calcium signaling pathway (Figure 7c). These pathways are critical and have a profound role in growth and metabolism, highlighting the importance of miR-210 in prenatal development. miR-199 downregulates fatty acid transport protein 1 (*fatp1*) and suppresses adipogenic trans-differentiation in C212 myoblasts [41]. A recent study reported that miR-199 is detectable in peripheral blood, and promotes muscle regeneration and myogenic differentiation [42]. Our results showed that miR-199 can potentially target 25 genes in LDM significantly correlated to fetal weight at 63 dpc. Collectively, these findings make miR-199 a potential candidate for non-invasive diagnosis of prenatal fetal health and growth. In C2C12 cells, miR-29c overexpression promotes cell differentiation, whereas overexpression of miR-29c in tibialis anterior muscles of mice increases muscle mass due to increased satellite cell proliferation and differentiation [43]. Members of miR-29 family also target myogenin, serine/threonine kinase 3 (*AKT3*), and atrophy-related genes, such as muscle RING-finger protein-1 (*MuRF1*), atrogin-1, and histone deacetylase 4 (*HDAC4I*) [43,44,45]. miR-449 targets jagged canonical notch ligand 1 (*Jag1*) in C2C12 cells, and regulates the insulin signaling pathway [46]. miR-24 is a key regulator of myogenesis and promotes myogenic differentiation of bovine skeletal muscle-derived progenitor cells by targeting activin receptor type 1B (*ACVR1B*) [47].

In addition to eight miRNAs upregulated in IUGR as well as correlated to fetal weight at 63 dpc, five other miRNAs (miR-140, miR-27, miR-181, miR-186, and miR-99) were also significantly correlated to fetal weight in the current study. Interestingly, these miRNAs are also involved in skeletal muscle growth and development. miR-140 is downregulated in the skeletal muscle of fast-growing fish, and is associated with fish body growth [48]. miR-27 is highly expressed in differentiating skeletal muscle cells and promotes myogenesis of pig muscle satellite cells by targeting MyoD family inhibitor (*MDFI*) [49,50]. miR-181 promotes muscle cell differentiation by targeting homeobox protein homeobox A11 (*HOX-A11*), which is a repressor of the differentiation process [51]. miR-186 is also an important myogenic miRNAs, which inhibits the differentiation of C2C12 cells and primary muscle cells by targeting myogenin [52]. miR-99 targets myotubularin-related protein 3 (*MTMR3*) and regulates chicken skeletal muscle satellite cells’ proliferation and differentiation [53]. Involvement of these miRNAs in muscle growth explains the significant correlation between the expression of these miRNAs in LDM and fetal weight in pigs at 63 dpc. We suggest that the miRNAs significantly correlated to fetal weight play an important role in regulating prenatal muscle growth and muscle mass, which can be one of the factors affecting postnatal health and carcass quality.

We also found 1315 genes significantly correlated to fetal weight at 63 dpc. The downstream analysis of these genes showed their enrichment in KEGG pathways and biological processes critical for growth and metabolism. Some of the enriched KEGG pathways important for growth and metabolism included the calcium signaling pathway, insulin signaling pathway, estrogen signaling pathway, growth hormone synthesis, secretion and action, parathyroid hormone synthesis, secretion and action, aldosterone synthesis and secretion, glucagon signaling pathway, and thyroid hormone synthesis. Although all of these hormonal pathways are well known for their role in growth and metabolism, the growth hormone and thyroid hormones are the most important players in fetal growth. Growth hormone is the key hormone for fetal development and is involved in growth and development of all tissues in the body, including skeletal muscles [54]. Thyroid hormones are critical for normal fetal growth and metabolism and also facilitate the terminal differentiation of different fetal tissues [55]. Thyroid hormones also regulate important metabolic pathways, such as glucose metabolism, lipolysis, and regulation of body weight [56]. Moreover, the genes correlated to fetal weight significantly enriched several biological processes critical for growth and development, including the generation of neurons, Wnt signaling pathway, bone mineralization, artery development, mesoderm formation, sprouting angiogenesis, transforming growth factor β receptor signaling, and insulin-like growth factor receptor signaling pathway.

We also performed an integrative analysis of 13 miRNAs and 1315 genes significantly correlated to fetal weight at 63 dpc. Out of 1315 genes, 135 genes were negatively correlated as well as identified as potential targets of 13 miRNAs. These target genes significantly enriched important KEGG pathways, including glycolysis/gluconeogenesis, apoptosis, insulin secretion and insulin signaling pathway, glucagon signaling pathway, and pyruvate metabolism. Moreover, 135 target genes also enriched important biological processes, including ATP generation from ADP, ATP metabolic process, cellular ketone metabolic process, cellular aldehyde metabolic process, heart development, blastocyst formation, and maternal placenta development. The data from enrichment analysis further showed that TEA domain transcription factor 4 (*TEAD4*) and storkhead box 2 (*STOX2*) are involved in important biological processes, including blastocyst formation, female pregnancy, maternal placental development, and regulation of stem cell population (Table S6). *TEAD4* was also identified as a potential target of miR-27, whereas *STOX2* can be potentially targeted by miR-24 (Table S3). Similarly, phosphoglycerate mutase 2 (*PGAM2*) regulates important KEGG pathways, including the glucagon signaling pathway, glycine, serine and threonine metabolism, and glycolysis/gluconeogenesis (Table S5). Our data showed that *PGAM2* can be potentially targeted by miR-140, miR-199, miR-24, miR-27, and miR-449.

In this study, we identified miRNA-mRNA networks regulating prenatal fetal growth and development in the pig. This data will be useful for future studies aimed to understand the role of individual miRNAs or genes in skeletal muscle development and fetal growth. Out of 1315 genes in LDM significantly correlated to fetal weight, only 135 were negatively correlated and identified as potential targets of 13 miRNAs. This data suggests that, although miRNAs have emerged as major regulators of almost all biological processes, not all the genes correlated to fetal weight are directly regulated by miRNAs but could be indirectly regulated by miRNAs or working independent of miRNA regulation. Enrichment of these genes in important metabolic pathways shows that a disrupted expression of these genes during the prenatal period can have long-lasting effects even after birth, as suggested in Barker’s hypothesis [57]. Based on the fact that miRNAs of fetal origin can be detected in maternal circulation [58,59,60,61], we suggest that fetal weight-correlated miRNAs in LDM can potentially serve as biomarkers of fetal health and growth. Further studies are needed to explore the roles of these miRNAs in fetal growth and their potential to be used as biomarkers of fetal health.

## Data Availability

The data (figures and table) used to support the findings of this study are included within the article. The expression data are available in the Gene Expression Omnibus public repository with the GEO accession number GSE162754 and GSE162755.

## Supplementary Materials

The following are available online at https://www.mdpi.com/article/10.3390/genes12081264/s1, Table S1: miRNAs, Table S2: mRNAs, Table S3: miRNA-mRNA networks, Table S4: All genes enrichment.

## Abbreviations

LDM	Longissimus dorsi muscle
dpc	Days post conception
IUGR	Intrauterine growth restriction
miRNA	MicroRNA
DL	German Landrace
Pi	Pietrain
