# Erratum: Ali et al. In Utero Fetal Weight in Pigs Is Regulated by microRNAs and Their Target Genes. *Genes* 2021, *12*, 1264

**DOI:** 10.3390/genes12101600

**Published:** 2021-10-12

**Authors:** Asghar Ali, Eduard Murani, Frieder Hadlich, Xuan Liu, Klaus Wimmers, Siriluck Ponsuksili

**Affiliations:** 1 Leibniz Institute for Farm Animal Biology, Institute for Genome Biology, Wilhelm-Stahl-Allee 2, 18196 Dummerstorf, Germany; ali@fbn-dummerstorf.de (A.A.); murani@fbn-dummerstorf.de (E.M.); hadlich@fbn-dummerstorf.de (F.H.); liu.xuan@fbn-dummerstorf.de (X.L.); wimmers@fbn-dummerstorf.de (K.W.); 2 Faculty of Agricultural and Environmental Sciences, University Rostock, 18059 Rostock, Germany

The authors wish to make the following correction to their paper published in *Genes* [1]:

## 1. The Original Article Mistakenly Stated the Following on Page 3

In the current study, the transcriptomic data from LDM of all 118 F2 fetuses were analyzed to identify miRNAs and genes correlated to fetal weight at 63 dpc. Animal care and tissue collection procedures were approved by the Animal Care Committee of the State Mecklenburg-Western Pomerania, Germany (State Office Agriculture, Food Safety and Fishery; LALLF M-V/TSD/7221.3-2.1-010/03), and husbandry and slaughter conformed to the German Law of Animal Protection. The experimental protocol was approved by the Animal Care Committee of the Leibniz Institute for Farm Animal Biology, Dummerstorf, Germany.

## 2. This Should Be Replaced with

In the current study, the transcriptomic data from LDM of all 118 F2 fetuses were analyzed to identify miRNAs and genes correlated with fetal weight at 63 dpc. The procedures for animal care and tissue collection were reviewed and approved by the Animal Care Committee of the Leibniz Institute for Farm Animal Biology (FBN) (2021010_30_A14_töt). All procedures complied with national animal welfare law and EU directive 86/609/EEC for animal experiments.

The authors would like to apologize for any inconvenience caused to readers. This change does not affect the scientific conclusions of the article. The manuscript will be updated, and the original will remain online on the article’s webpage, with a reference to this Erratum notice.
